# A Study on the Assessment of Anxiety and Its Effects on Students Taking the National Eligibility cum Entrance Test for Undergraduates (NEET-UG) 2020

**DOI:** 10.7759/cureus.44240

**Published:** 2023-08-28

**Authors:** SreeRam Thiriveedhi, Achyuth Myla, CV Priya, Keerthana Vuppuluri, Phanindra Dulipala, Vijaya Krishna Prasad Vudathaneni

**Affiliations:** 1 Department of Community Medicine, Katuri Medical College and Hospital, Guntur, IND; 2 Department of Community Medicine, Government Medical College, Machilipatnam, IND; 3 Department of Internal Medicine, Albert Einstein College of Medicine, New York, USA

**Keywords:** stress, neet-ug, test anxiety, students, mental health

## Abstract

Background

Test anxiety is a major, often overlooked, mental health concern among students. We live in a society that decides a person's future by their performance in an examination. In our country, issues about test anxiety, academic-related depression, and stress are less discussed. Most of the institutions don’t guide students with stress management. The present study was conducted to measure the anxiety levels and their effect on the mental health of students taking the National Eligibility cum Entrance Test for Undergraduates (NEET-UG) 2020.

Methods

A cross-sectional study was done among 200 students of a private junior college taking the NEET-UG 2020 exam using the Westside Test Anxiety Scale questionnaire. The results obtained were analyzed by appropriate statistical tests using SPSS Statistics version 25 (IBM Corp. Released 2017. IBM SPSS Statistics for Windows, Version 25.0. Armonk, NY: IBM Corp.).

Results

Out of 200 students, the results showed that, overall, 151 (75.5%) were stressed out before the exam, and 49 (24.5%) were not stressed out. This study revealed that the majority of the students didn't receive any professional help to combat their mental health problems or any professional counseling to improve their morale.

Conclusion

It is evident from the results of the study that the majority of the students faced difficulties in their academic performance due to test anxiety, and the effects were prominent irrespective of the age and gender of the students. Professional psychological counselors, guidance, and the availability of healthcare professionals in institutions to address the mental health needs of students will yield better outcomes.

## Introduction

Test anxiety is a major, often overlooked, mental health concern among students. Test anxiety can be defined as a “multidimensional construct combining with worry, emotionality, and interference, fear of failure, self-esteem and lack of confidence” [1]. It is a “combination of physiological over-arousal, tension, and somatic symptoms, along with worry, dread, fear of failure, and catastrophizing, that occur before or during test situations” [2]. Higher anxiety levels can have debilitating effects on students and can cause detrimental effects when they undertake any examination [3].

The National Eligibility cum Entrance Test (NEET) is an entrance examination conducted for entry into medical colleges in India. Every year, nearly 13-14 lakh students register for this examination, but with the limited number of medical seats in our country, the stakes are high for those who take the test, creating pressure on students [4]. This academic pressure may manifest as anxiety in the students during the examination [5]. These higher stakes create a pressure-laden atmosphere around the students, affecting their mental health and leading to its deterioration [6].

In our country, issues about test anxiety, academic-related depression, and stress are less discussed. The majority of schools do not offer students strategies for coping with test anxiety and mental health issues. They even divide the pupils into groups based on the results of the tests they conduct. These techniques place even greater pressure on the students to maintain their performance levels. Not addressing these predicaments could cause serious problems or perhaps put students in a position where they might end their own lives.

Not much research has been done regarding test anxiety in our country. This study is focused on the students taking the NEET-UG 2020. By this, we may be able to determine the extent of the harm that test anxiety can cause and also direct us to take the appropriate steps to safeguard the future of the students who will become tomorrow's citizens. The main objective of this study was to measure the students' anxiety levels and their effects on their sleep and mental status and assess the knowledge of various management methods of test anxiety.

## Materials and methods

This cross-sectional study was conducted among students preparing to attend the NEET-UG 2020 examination (pre-medical entrance test in India) using convenience sampling. Students who were going to take the NEET-UG 2020 for the first time were included in the study. Students who already took the NEET and will retake it in 2020 were excluded from this study.

A self-designed, semi-structured questionnaire was used to collect the data, which consists of three sections. Section one consisted of the standard Westside Test Anxiety Scale questionnaire [7]. The students responded to a 10-item questionnaire on a 5-point scale in section one. The rating of a question was from 1 to 5, 1 being not at all or never accurate and 5 being extremely or always true. The sum of the ratings of the 10 questions was divided by 10, which gave us the final test anxiety score. In section two, the students were inquired about their sleep and state of mind through “yes-or-no” questions. The students' responses to those questions were analyzed and correlated to their anxiety levels. In section three, the students were implored upon the subject of anxiety management techniques, the source of this management knowledge, whether students were implementing these techniques or not, and their efficiency in reducing test anxiety along with their performance in the tests.

Data was collected using a questionnaire given to the students attending the NEET-UG 202, consisting of three sections. The first section covered questions regarding the condition of the students before the entrance test and during an identical mock test. The students were enquired about their mental status during the test. The Westside Test Anxiety Scale was used for this purpose [7]. The second section contained questions related to their sleep and mood before the exam. The third section addressed questions related to their management of anxiety. The last two sections contained self-prepared questions that targeted the mental condition of the students before the exam.

The study was done after the required ethical certificates, assent, and consent forms were approved by the Institutional Ethics Committee of a tertiary-care teaching hospital, Katuri Medical College and Hospital (IEC No: 25/2020). The confidentiality of the students was maintained throughout the study and was given the highest significance.

The collected data was analyzed using descriptive statistics containing means, percentages, and standard deviations. The percentage of students with sleep difficulties and high anxiety levels was also interpreted. The data was evaluated using Microsoft Excel (Microsoft Corp., Redmond, WA, USA) and SPSS Statistics version 25 (IBM Corp. Released 2017. IBM SPSS Statistics for Windows, Version 25.0. Armonk, NY: IBM Corp.), and appropriate statistical methods like the chi-square test were used. Anxiety levels in male and female students were evaluated separately.

## Results

Demographic characteristics

**Figure 1:**
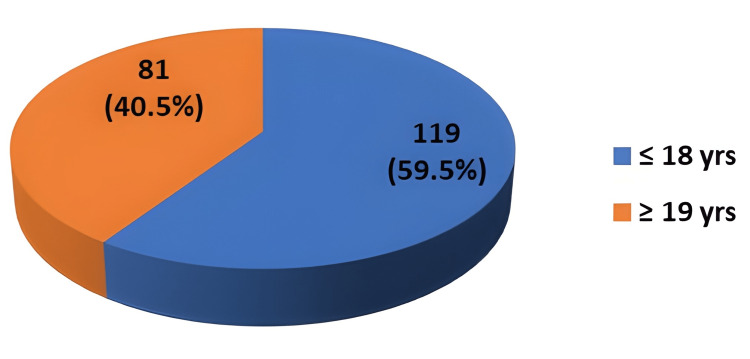
Distribution of students according to age (n=200)

In this study, the students were distributed as ≤18 years old and ≥19 years old. The age distribution of the study subjects is shown in Figure 1. Eighty-one (40.5%) students belong to the ≤18 years and 119 (59.5%) belong to the ≥19 years age category. The mean age of the study population was 18.33± 1.152.

**Figure 2:**
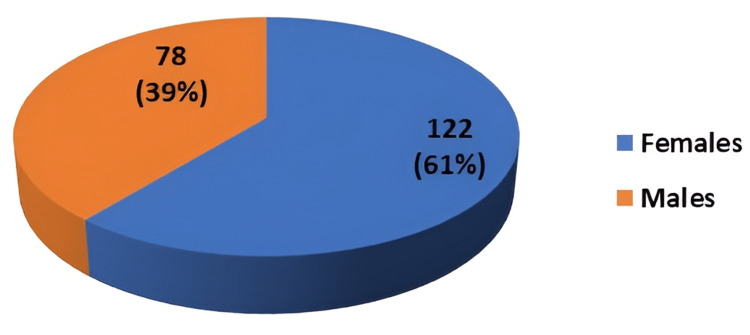
Distribution of students according to gender (n=200)

The gender distribution is shown in Figure 2. In the study, out of the 200 students, 122 (61%) were females and 78 (39%) were males.

Anxiety levels among students

**Figure 3:**
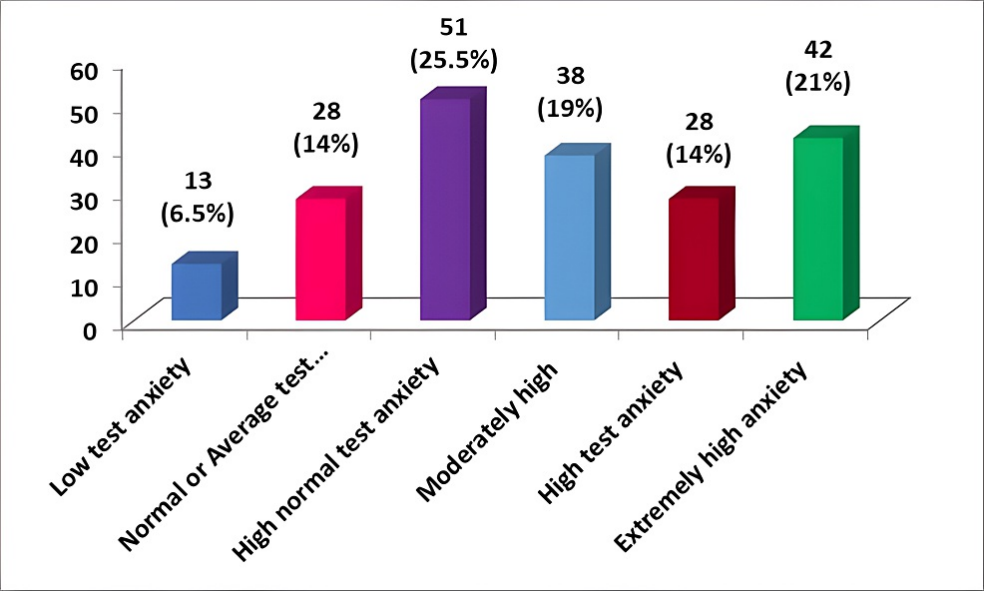
Grading of test anxiety among students (n=200)

Figure 3 represents the anxiety grading among the study subjects. This study of test anxiety assessment showed that 51 (25.5%) of the students were in high normal test anxiety level, 38 (19%) were in moderately high anxiety, 28 (14%) were in high test anxiety, and 42 (21%) were in extreme test anxiety. Overall, 13 (6.5%) students had no anxiety, while 108 (54%) of the students were in the range of moderately high to extremely high test anxiety.

Table 1 interprets the relationship between anxiety grading and gender. This study revealed that 6 (4.9%) females and 7 (9.0%) males showed comfortably low test anxiety, whereas 31 (25.4%) females and 20 (25.6%) males showed high normal test anxiety, 26 (21.3%) females and 12 (15.4%) males showed moderately high anxiety, 17 (13.9%) females and 11 (14.1%) males showed high test anxiety, and 22 (18.0%) females and 20 (25.6%) males showed extremely high anxiety levels. A non-significant relationship was observed between gender and anxiety levels.

**Table 1:** Relationship between gender and anxiety grading (n=200) Pearson chi-square 4.678, p-value 0.456 (non-significant), 95% CI, and a p-value <0.05 (statistically significant)

Gender	Anxiety grading						Total
	Comfortably low-test anxiety	Normal or average test anxiety	High normal test anxiety	Moderately high	High test anxiety	Extremely high anxiety	
Female	6 (4.9%)	20 (16.4%)	31 (25.4%)	26 (21.3%)	17 (13.9%)	22 (18.0%)	122 (61%)
Male	7 (9.0%)	8 (10.3%)	20 (25.6%)	12 (15.4%)	11 (14.1%)	20 (25.6%)	78 (39%)

Effect of anxiety on mental status and sleep

**Figure 4:**
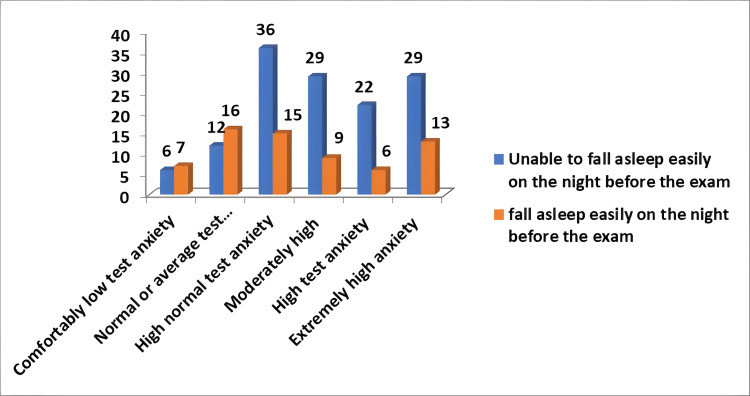
Effect of anxiety on sleep the night before the exam (n=200)

Out of 200 study subjects, test anxiety had an effect on sleep for 136 (68%) people the night before the exam, while it had no effect on sleep for 64 (32%). It was a statistically significant relationship with a Pearson chi-square value of 13.500 and a p-value of 0.019 (Figure 4).

Table 2 interprets the relationship between test anxiety and stress before the exam. In our study, the comfortably low test anxiety group (92.3%) was less stressed out compared to others. Overall, 153 (76.5%) were stressed out before the exam, and 47 (23.5%) were not stressed out. It was a statistically significant relationship.

**Table 2:** Relationship between anxiety grading and stress before the exam (n=200) Pearson chi-square 18.579, p-value 0.002 (statistically significant)

Test anxiety grading	Stressed out before the exam		Total
	No	Yes	
Comfortably low test anxiety	7 (53.8%)	6 (46.2%)	13 (6.5%)
Normal or average test anxiety	12 (42.9%)	16 (57.1%)	28 (14%)
High normal test anxiety	13 (25.5%)	38 (74.5%)	51 (25.5%)
Moderately high	4 (10.5%)	34 (89.5%)	38 (19%)
High test anxiety	4 (14.3%)	24 (85.7%)	28 (14%)
Extremely high anxiety	7 (16.7%)	35 (83.3%)	42 (21%)

Student knowledge of the management of test anxiety

Table 3 shows the relationship between grade and knowledge of techniques of anxiety management. Students in the comfortably low test anxiety group knew more (13.5%) anxiety management techniques when compared to students who were more anxious (69.1%).

**Table 3:** Relationship between anxiety grading and techniques of anxiety management (n=200) Pearson chi-square 17.7, p-value 0.003 (statistically significant)

Test anxiety grading	Do you know any of the anxiety management techniques?		Total
	No	Yes	
Comfortably low test anxiety	2 (15.4%)	11 (84.6%)	13 (6.5%)
Normal or average test anxiety	14 (50.0%)	14 (50.0%)	28 (14%)
High normal test anxiety	25 (49.0%)	26 (51%)	51 (25.5%)
Moderately high	27 (71.1%)	11 (28.9%)	38 (19%)
High test anxiety	16 (57.1%)	12 (42.9%)	28 (14%)
Extremely high anxiety	30 (71.4%)	12 (28.6%)	42 (21%)

The relationship between the grade of anxiety and the implementation of management techniques is shown in Table 4. This study revealed that normal or high normal test anxiety students knew more about anxiety management and were able to control it. Most of the students do not even know about the anxiety management techniques. However, if they were implemented, there would be a significant result.

**Table 4:** Relationship between anxiety grading and implementing anxiety management techniques (n=200) Pearson chi-square 25.416, p-value 0.005 (statistically significant)

Test anxiety grading	If you know the techniques of anxiety management, are you implementing them?			Total
	I don't know any of the techniques	No	Yes	
Comfortably low test anxiety	2 (15.4%)	3 (23.1%)	8 (61.5%)	13 (6.5%)
Normal or average test anxiety	14 (50.0%)	1 (3.6%)	13 (46.4%)	28 (14%)
High normal test anxiety	25 (49.0%)	8 (15.7%)	18 (35.3%)	51 (25.5%)
Moderately high	27 (71.1%)	2 (5.3%)	9 (23.7%)	38 (19%)
High test anxiety	16 (57.1%)	4 (14.3%)	8 (28.6%)	28 (14%)
Extremely high anxiety	30 (71.4%)	7 (16.7%)	5 (11.9%)	42 (21%)

## Discussion

This study showed that 51 (25.5%) of the students had a high normal test anxiety level, 38 (19%) had moderately high anxiety, 28 (14%) had high test anxiety, and 42 (21%) had extreme test anxiety. Overall, 13 (6.5%) students had no anxiety, while 159 (79.5%) had high to extreme anxiety. It is concerning that an increasing number of students are suffering from test anxiety. This study coincided with the research done by Shrivastava et al., who indicated that more than 50% of the study group is suffering from anxiety, which ranges from moderate to severe [8]. Many other studies conducted by Schouwenburg, Sarason, and Mandler and Sarason showed that test anxiety is a problem that needs to be dealt with with immediate attention [2,9,10]. The results also concurred with the basic findings from the study by Singh et al. that showed that most students suffer from anxiety [11].

In the present study, the comfortably low test anxiety group (92.3%) was less stressed out than others. Overall, 153 (76.5%) were stressed out before the exam, and 47 (23.5%) were not stressed out. About 83.3% of the extremely test anxious students, 85.7% of the highly test anxious students, and 89.5% of moderately test anxious students were stressed out before the exam. Low test anxious students were stressed out. The majority of the students were also depressed before the exam. This reflects the fact that test anxiety has an effect on the state of a person, and this needs immediate attention and consideration. The results also matched up with the study done by Satish Kumar et al. that revealed that 81.6% of respondents have at least one of depression, anxiety, and stress [12], the study done by Jayanthi et al. that showed that there is a positive relationship between the level of depression and the level of academic stress [13], and the study done by Iqbal et al. using the Depression Anxiety and Stress Scale score that showed a correlation between the overall low mental health and academic underachievement [14]. According to a study by Depreeuw and De Neve, test anxious students are also mentally affected and exhibit psychosomatic symptoms [6].

In this study, among 200 students, 61% were females. This study revealed that six (4.9%) females and seven (9.0%) males had comfortably low test anxiety, whereas 31 (25.4%) females and 20 (25.6%) males had high normal test anxiety, 26 (21.3%) females and 12 (15.4%) males had moderately high anxiety, 17 (13.9%) females and 11 (14.1%) males had high test anxiety, and 22 (18.0%) females and 20 (25.6%) males had extremely high anxiety levels. This study showed that females are more anxious than males but only have average and moderately high anxiety levels. Males, on the other hand, have high normal test anxiety, high test anxiety, and extremely high test anxiety. There was not much variation in the percentage of males and females with high normal and high test anxiety levels, and there was no statistically significant relationship found between gender and test anxiety. However, based on the results, we can assume that females face test anxiety within moderate limits, whereas males are either highly anxious or low anxious. This may be due to the fact that a slightly higher number of females (45.1%) in this study group knew about the anxiety management techniques, and 43.6% of them found them efficient. Compared to these results, 60.3% of males did not know any techniques to reduce anxiety, and only 41.9% of those who knew found them useful. Other previous studies showed a marked difference in anxiety levels faced by females. They discovered that females feel more test anxiety than males [12,14,15]. One study attributed these findings to the psychological differences between men and women, where females are more sensitive and more likely to articulate worries and emotions [16]. The fact that females need to be more accomplished than males in a respective field to receive equal recognition as their male equivalents is an important factor to keep in mind because this is one of the reasons why they may feel more anxious. The reason for the insignificant difference between anxiety levels in males and females may be due to the fact that this study group was faced with almost the same amount of pressure. The future of students depends on their performance in this entrance test.

In this study, 136 (68%) students could not sleep the night before the exam, whereas 64 (32.0%) did not affect sleep, which is a statistically significant relationship. These results were consistent with the study done by Pensuksan et al. that suggested that students classified as poor-quality sleepers had a high prevalence of depression of about 45.5%. Poor-quality sleepers reported moderate depression (26.7%), moderate anxiety (29.3%), and moderate stress (22.9%) [17]. Anxiety and depression lead to less sleep quality and, therefore, impact the students' performance in their day-to-day tasks and academic activities. The majority of students with high test anxiety found it difficult to fall asleep easily the night before their exams.

This study revealed that the majority of the students didn't receive any professional help to combat their mental health problems or professional counseling to improve their morale. More than 70% of the students said they didn't receive any help from the side of the college. Only 43% of the students knew about any anxiety management techniques, and only 30.5% implemented them. Among them, only 43% of the students expressed that they were effective. Even though some students implemented these techniques, whose source is unknown, it was only partially effective in high-grade anxiety students. It is to be noted that the majority of the students with comfortably low test anxiety to normal test anxiety grades were aware of anxiety reduction techniques. Furthermore, the majority of the students in the comfortably low-test anxiety grade expressed that these techniques did help them overcome their anxiety. This shows that other techniques are needed for high-grade anxiety students to battle their anxiety issues. In a study done by Shrivastava et al., depression and anxiety are statistically significant in students who never got support as compared to students who always got support from faculty [8]. This shows that any help from the faculty or college can reassure and relieve students.

Limitations

The study group consisted of limited students from only one private institution. Therefore, other institutions’ students' conditions and status were unknown. Limited research is present right now regarding test anxiety in our country, and hence, extensive research in this particular area must be done across the country in many institutions to identify and analyze factors that contribute to test anxiety. Factors that may deteriorate the mental health condition of students must be identified and dealt with. The government should implement policies and programs to provide psychological counseling and guidance, and if required, help from healthcare professionals should be made available to students at the institutional level. Further research must also include students from different age groups. Comparative studies between urban and rural institutions regarding test anxiety must be done to know the factors affecting students in other regions.

## Conclusions

It is evident from the results of the study that the majority of students are facing difficulties in their academic performance due to test anxiety and its debilitating effects. Associated difficulties like depression, stress, and loss of quality sleep also contribute to students' underperformance in the exams. The effects are prominent irrespective of the age and gender of students. The majority of students complain of mental health problems due to anxiety and do not know any management techniques. The most alarming finding is that, in most cases, no assistance is available to students in overcoming their fears and barriers. The immense pressure from parents and colleges, including the pressure students put on themselves, will shatter them. They are the medical care professionals of the future. Hence, preserving their health is of utmost importance as they will preserve the health of the masses in the future. The most alarming finding in this study is how little help students receive in resolving their worries and challenges. Professional help must be accessible to students to combat test anxiety. Anxiety management techniques must be taught to students so that they can implement them. Psychological counseling must be provided to overcome their fears and achieve maximum willpower and concentration.
